# Supplementary material for: A Comprehensive Analysis of Transcriptomics and Proteomics Elucidates the Cold-Adaptive Ovarian Development of Eriocheir sinensis Farmed in High-Altitude Karst Landform
Source: Genes (Basel). 2025 Sep 6;16(9):1048. doi: 10.3390/genes16091048 (PMC12469982; doi:10.3390/genes16091048)
Supplement: Supplementary file 1 [file genes-16-01048-s001.zip › genes-3842908-supplementary.pdf]

A

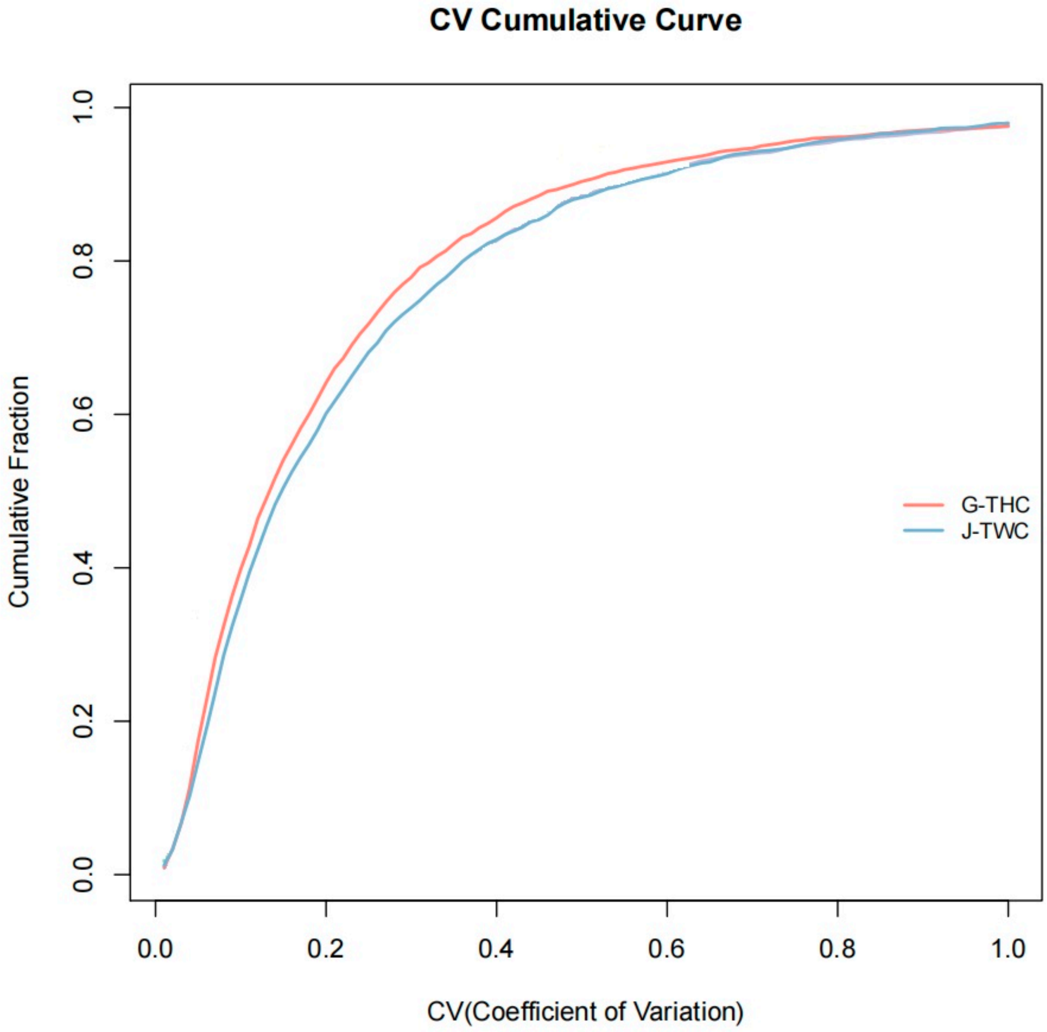
CV Cumulative Curve
Cumulative Fraction
0.0
0.2
0.4
0.6
0.8
1.0
CV(Coefficient of Variation)
G-THC
J-TWC

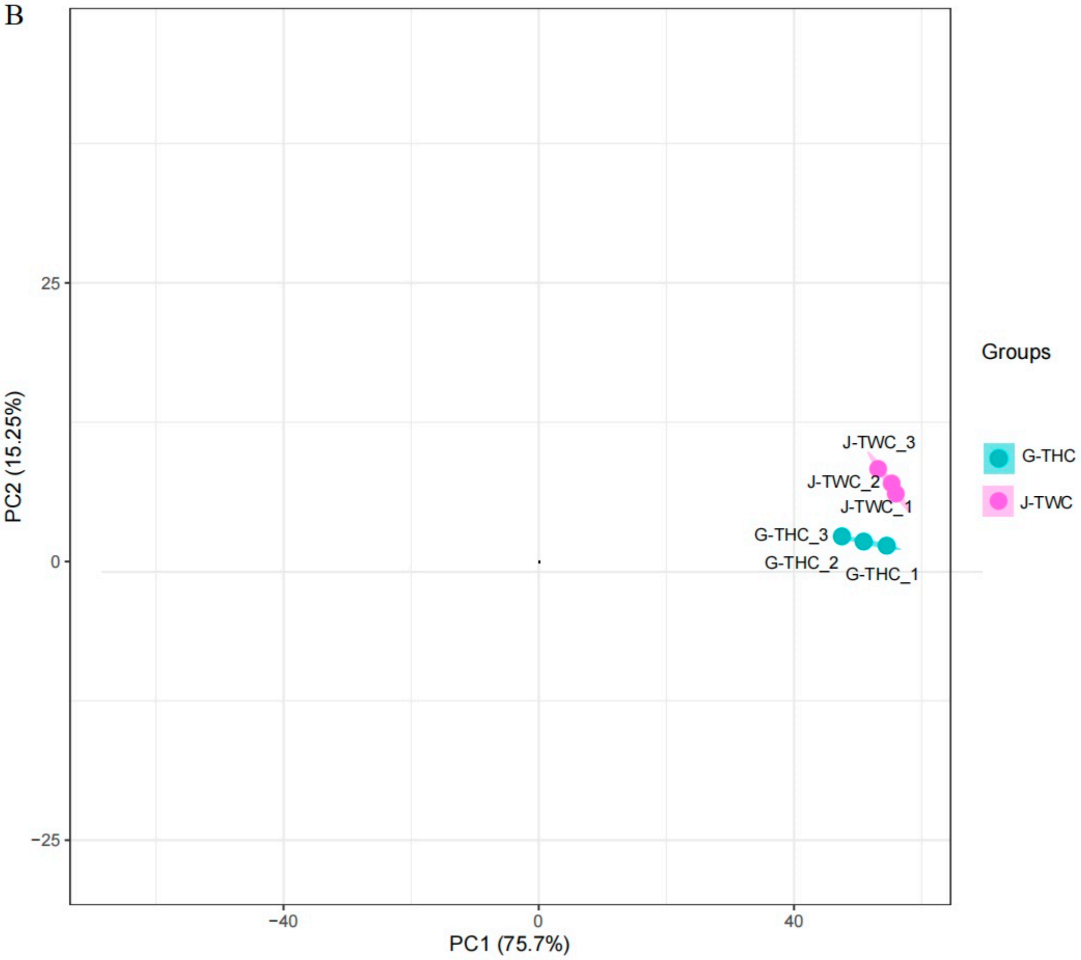

Figure S1. Quantitative analysis of the detected proteins was performed using proteomics. This analysis included coefficient of variance analysis (A) and principal component analysis (B)

A

B

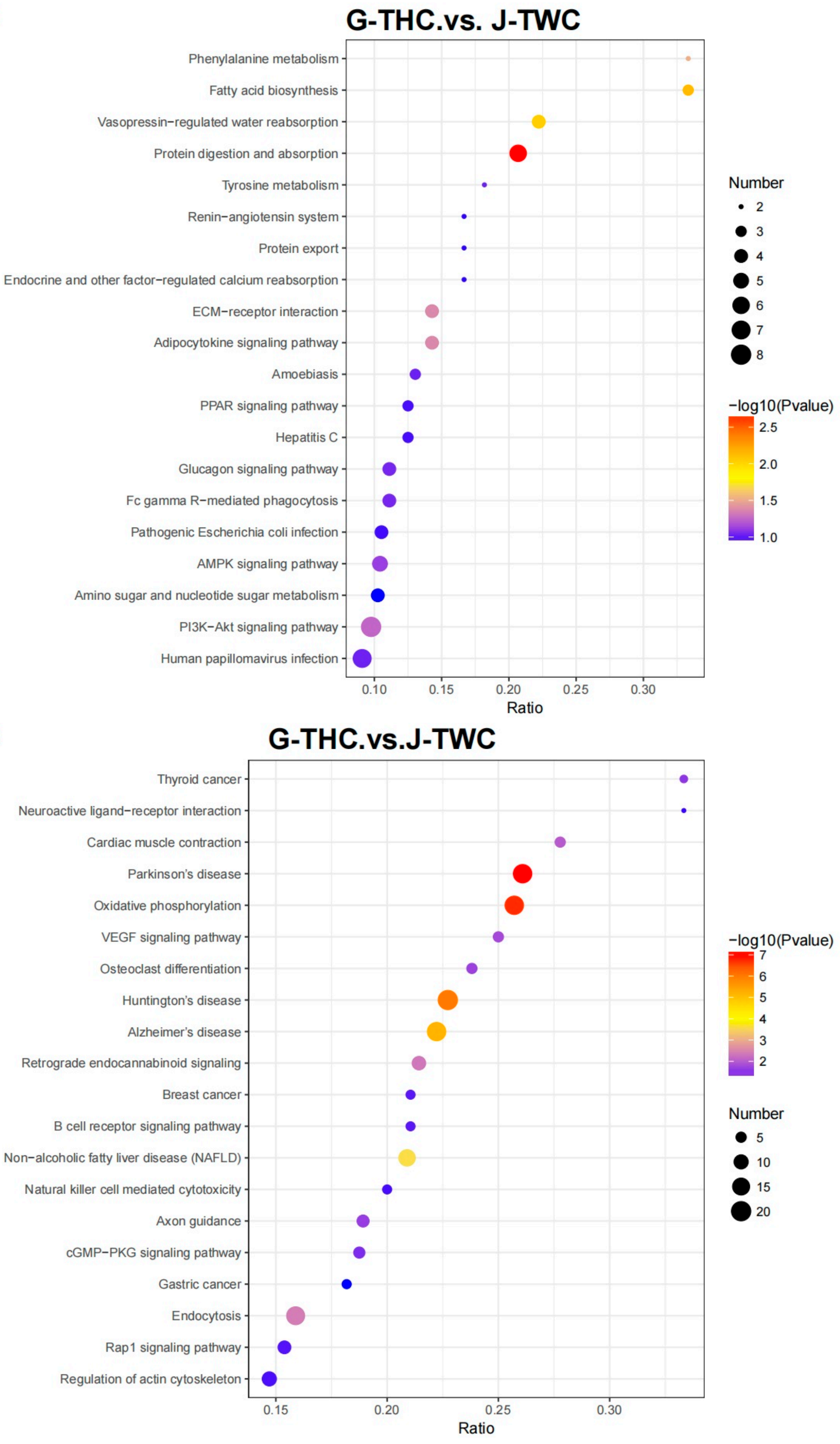
G-THC.vs. J-TWC
Phenylalanine metabolism
Fatty acid biosynthesis
Vasopressin−regulated water reabsorption
Protein digestion and absorption
Tyrosine metabolism
Renin−angiotensin system
Protein export
Endocrine and other factor−regulated calcium reabsorption
ECM−receptor interaction
Adipocytokine signaling pathway
Amoebiasis
PPAR signaling pathway
Hepatitis C
Glucagon signaling pathway
Fc gamma R−mediated phagocytosis
Pathogenic Escherichia coli infection
AMPK signaling pathway
Amino sugar and nucleotide sugar metabolism
PI3K−Akt signaling pathway
Human papillomavirus infection
0.10 0.15 0.20 0.25 0.30
Ratio
Number
2 3 4 5 6 7 8
−log10(Pvalue)
2.5 2.0 1.5 1.0
G-THC.vs.J-TWC
Thyroid cancer
Neuroactive ligand−receptor interaction
Cardiac muscle contraction
Parkinson's disease
Oxidative phosphorylation
VEGF signaling pathway
Osteoclast differentiation
Huntington's disease
Alzheimer's disease
Retrograde endocannabinoid signaling
Breast cancer
B cell receptor signaling pathway
Non−alcoholic fatty liver disease (NAFLD)
Natural killer cell mediated cytotoxicity
Axon guidance
cGMP−PKG signaling pathway
Gastric cancer
Endocytosis
Rap1 signaling pathway
Regulation of actin cytoskeleton
0.15 0.20 0.25 0.30
Ratio
−log10(Pvalue)
7 6 5 4 3 2
Number
5 10 15 20

Figure S2. The up-regulated (A) and down-regulated (B) DEPs in the comparison of G-THC and J-TWC were subjected to the KEGG function classification.
